# Effects of neflamapimod (p38α kinase inhibitor) on clinical progression in patients with dementia with Lewy bodies (DLB) without Alzheimer’s disease (AD) Co‐Pathology

**DOI:** 10.1002/alz70861_108769

**Published:** 2025-12-23

**Authors:** Lawrence S. Honig, Stephen N Gomperts, John‐Paul Taylor, Niels D. Prins, Amanda Gardner, Kelly Blackburn, John J Alam, James E. Galvin

**Affiliations:** ^1^ Columbia University Irving Medical Center, New York, NY USA; ^2^ Massachusetts General Hospital, Boston, MA USA; ^3^ Translational and Clinical Research Institute, Newcastle University, Newcastle upon Tyne UK; ^4^ Brain Research Center, Amsterdam Netherlands; ^5^ CervoMed Inc, Boston, MA USA; ^6^ University of Miami Miller School of Medicine, Boca Raton, FL USA

## Abstract

**Background:**

RewinD‐LB phase 2b study (NCT05869669) was initiated to confirm phase 2a results in DLB in which neflamapimod demonstrated positive effects on multiple clinical endpoints, most prominently in patients without evidence of AD co‐pathology (assessed by plasma ptau181).

**Method:**

159 participants with DLB by consensus criteria, screening plasma ptau181<2.4 pg//mL (i.e., without AD co‐pathology) were enrolled in a 16‐week randomized (1:1), placebo‐controlled study (“Initial Phase”), with a 32‐week neflamapimod‐only Extension. Unfortunately, pharmacokinetic measurements in the Initial Phase showed that expected plasma drug concentrations were not achieved. With introduction during the Extension of a new batch of capsules that achieved the targeted plasma drug concentrations, the effects of neflamapimod treatment with New Capsules (*N* =94; active drug arm) during the 1st 16 weeks of the Extension could be compared against outcomes in participants who continued to receive Old Capsules (*N* =55), which served as a lower drug level control arm. Endpoints were analyzed by linear mixed effects model for repeated measures (MMRM), except for CGIC (analyzed by Mann‐Whitney).

**Result:**

During the initial phase, there were no discernible differences on any of the endpoints between neflamapimod and placebo. However, during the Extension phase, with New Capsules there was improvement on change in CDR‐SB (*p* <0.001 vs. Old Capsules during Extension; *p* =0.003 vs. placebo utilizing all study data), improvement on ADCS‐CGIC (*p* =0.035 vs. Old Capsules during Extension; *p* =0.036 in a within‐participant comparison to placebo during the Initial Phase), and improvement vs. Old capsules during Extension on DCFS (fluctuations) and ISLT‐Recognition (working memory). There were also positive trends vs. Old Capsules on NPI‐12, TUG and UPDRS Part III (Motor); and a lower incidence of falls vs either Old Capsules or placebo. Discontinuation for adverse events was ≤ 4% in each phase.

**Conclusion:**

With achieving target plasma drug concentrations, there was evidence of neflamapimod having a meaningful impact on clinical progression, as assessed by the CDR‐SB and CGIC, in patients with DLB who do not have AD co‐pathology. The pattern of the effects across the full set of clinical endpoints evaluated are consistent with preclinical results in which neflamapimod beneficially impacts the disease process in the basal forebrain.

